# Dose–response of spinal manipulation for cervicogenic headache: study protocol for a randomized controlled trial

**DOI:** 10.1186/s12998-016-0105-z

**Published:** 2016-06-08

**Authors:** Linda Hanson, Mitchell Haas, Gert Bronfort, Darcy Vavrek, Craig Schulz, Brent Leininger, Roni Evans, Leslie Takaki, Moni Neradilek

**Affiliations:** Center for Spirituality and Healing, The University of Minnesota, 420 Delaware St SE C592, Minneapolis, MN 55455 USA; Center for Outcome Studies, The University of Western States, 2900 NE 132nd Ave, Portland, OR 97230 USA; Biostatistics, Clinical and Regulatory Affairs, Illumina, 5200 Illumina Way, San Diego, CA 92122 USA; Children’s Hospitals and Clinics of Minnesota, Pain, Palliative Care, and Integrative Medicine, 2525 Chicago Ave, Minneapolis, MN 55404 USA; The Mountain-Whisper-Light Statistics, 1827 23rd Ave East, Seattle, WA 98112 USA

**Keywords:** Cervicogenic headache, Spinal manipulation, Randomized controlled trial, Mixed methods, Chiropractic

## Abstract

**Background:**

Cervicogenic headache is a prevalent and costly pain condition commonly treated by chiropractors. There is evidence to support the effectiveness for spinal manipulation, but the dose of treatment required to achieve maximal relief remains unknown. The purpose of this paper is to describe the methodology for a randomized controlled trial evaluating the dose–response of spinal manipulation for chronic cervicogenic headache in an adult population.

**Methods/Design:**

This is a mixed-methods, two-site, prospective, parallel groups, observer-blind, randomized controlled trial conducted at university-affiliated research clinics in the Portland, OR and Minneapolis, MN areas. The primary outcome is patient reported headache frequency. Other outcomes include self-reported headache intensity, disability, quality of life, improvement, neck pain intensity and frequency, satisfaction, medication use, outside care, cervical motion, pain pressure thresholds, health care utilization, health care costs, and lost productivity. Qualitative interviews are also conducted to evaluate patients’ expectations of treatment.

**Discussion:**

With growing concerns regarding the costs and side effects of commonly used conventional treatments, greater numbers of headache sufferers are seeking other approaches to care. This is the first full-scale randomized controlled trial assessing the dose–response of spinal manipulation therapy on outcomes for cervicogenic headache. The results of this study will provide important evidence for the management of cervicogenic headache in adults.

**Trial registration:**

ClinicalTrials.gov (Identifier: NCT01530321)

## Background

Headaches are a common, disabling condition with a substantial public health and financial impact on society [1]. Approximately half to three quarters of the global population experiences a headache during their life [2], and the financial costs on society are huge. It is estimated that 157 million days of work are lost each year due to headaches, costing approximately $50 billion in work absenteeism and medical benefits [3]. Neck pain is common among headache sufferers [4], and secondary headache pain referred from a neck disorder is defined as cervicogenic headache (CGH) [5]. The point prevalence for CGH ranges from 0.4 to 4.6% [6–8] and up to 18% of the chronic headache population due to variation in its definition [9].

Because of the growing concerns about harmful side effects from over utilized pain medication [10], there is a great need to investigate effective, safe, and cost-effective complementary and integrative health therapies for headache conditions. About 34% of US adults use such therapies annually [11], many for headache and neck pain [12, 13]. Spinal manipulative therapy (SMT), a commonly used treatment for headache [4], is among the most common [14].

A growing body of literature supports the use of SMT for headache and no other intervention has been shown to be superior for the care of CGH [15–18]. Efficacy of SMT for the relief of chronic CGH has been summarized in systematic reviews [15, 19]; one review however found insufficient evidence to reach a conclusion [20]. Most randomized controlled trials found evidence of efficacy for SMT for CGH frequency, intensity and duration [4, 21–23], specifically compared to deep massage [24] and no treatment [25]. Despite this, there remains little consensus on what constitutes an appropriate dose of manipulation needed to achieve maximal benefit [26–30]. Preliminary studies suggest a dose–response relationship for SMT in the management of CGH [27, 29]. One pilot RCT (*n* = 24) examined differences between three doses: 3, 9, or 12 SMT visits and found preliminary benefits with larger doses [27]. A second (*n* = 80) compared 8 and 16 SMT visits and found clinically important differences between SMT and the control and small differences between the two doses for a number of headache outcomes [29]. This is the first full-scale randomized controlled trial to investigate this relationship.

### Study aims

The primary aim is to determine the effect of SMT visits on self-reported clinical outcomes and objective physical measures in 256 adults with chronic CGH (≥3 months), measured at 12 and 24 weeks. The primary outcome is patient-rated CGH frequency measured in days in the 4 weeks prior to these time points. Our hypothesis is that a greater number of SMT treatments leads to a greater reduction in CGH frequency. The secondary aim is to determine the cost-effectiveness and cost-utility of the number of SMT treatments for the care of chronic CGH. The tertiary aim is to assess the effects of expectations on outcomes using mixed-methods in order to gain a better understanding of how patients view their headaches and treatment.

## Methods/Design

### Study overview

This study is a two-site, prospective, parallel groups, observer-blinded, randomized controlled trial. The trial began in August 2012. Participants are recruited at Northwestern Health Sciences University in Bloomington, MN and the University of Western States in Portland, OR. Study treatments are provided within university-affiliated outpatient clinics. The study design is based on a previous pilot randomized controlled trial [29].

### Funding and ethical approval

The trial is funded by the National Institutes of Health National Center for Complementary and Integrative Health (R01AT006330). Ethical approval is granted by the Institutional Review Boards (IRB) at the two participating institutions (IRB20110127 and ID 1-98-10-11). The trial is registered on ClinicalTrials.gov (Identifier: NCT01530321), and informed consent is obtained from all participants.

### Recruitment

Potential subjects are recruited from Minneapolis, MN, Portland, OR and their surrounding metropolitan communities using multiple recruitment methods. These include systematic mailings of study post-card mailers, online advertisements (Craigslist, Facebook, news websites), local radio, newspaper and community postings, and referrals from community medical clinics.

### Study population

**Fig. 1:**
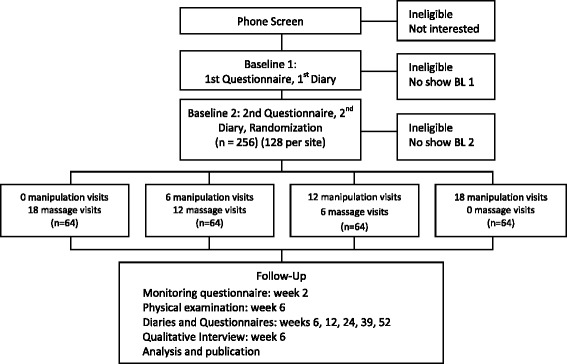
Participant flow

Adults, age 18 and older with a history of chronic CGH are eligible to participate. The eligibility criteria are described in Table 1. Participant flow (Fig. 1) data are recorded in accordance with the Consolidated Standards of Reporting Trials (CONSORT) [31] statement and will be reported with final study results.

**Table 1:** Inclusion and exclusion criteria

Inclusion criteria	Exclusion criteria
- 18 years of age and older - History of CGH ≥ 3 months at Baseline 1, ≥5 days of CGH per month - CGH intensity ≥3 (0–10 scale) - Cervical spine dysfunction (cervical joint tenderness and/or restricted segmental motion) - Clear temporal sequence linking the source of CGH to the neck: headache preceded by neck pain, stiffness, movement and/or awkward postures - English literate - Independent ambulation	- Other headaches within one year of enrollment (e.g. migraine occurring on >1 day per month in the last year, medication overuse, daily, cluster, temporomandibular joint dysfunction related headaches, sinus, posttraumatic, tumor and glaucoma related, occipital neuralgia, metabolic/toxic/substance abuse related). - Spinal manipulative therapy, massage or exercise therapy for neck pain or headaches in the previous 3 months. Any other types of care by a licensed provider in the previous month for headaches or neck pain - Contraindications to study treatments (e.g., inflammatory arthropathies, cervical instability, severe osteoporosis, vertigo, dizziness) - Daily prescription or nonprescription pain medication; corticosteroid use in previous month - Cancer in the past five years - Cardiovascular comorbidities (e.g., history of stroke, transient ischemic attacks, stage 2 hypertension, taking anticoagulant medication, syncope, myocardial infarction, hemophilia) - Neurological comorbidities (e.g., multiple sclerosis, ALS, Parkinson’s, myelopathy, seizures, cervical radiculopathy, herniated disc, thoracic outlet syndrome, brachial plexus neuropathy) - Spinal pathology (e.g., infection, tumor, fracture, diffuse idiopathic skeletal hyperostosis, degenerative joint disease, stenosis) - Involved in a research study about pain - Active or pending medical litigation, personal injury, workers compensation; disability compensation - Noncompliance with headache diary at baseline (<24/28 days completed); pre-randomization noncompliance - Pregnancy, trying to get pregnant, 3 months post-partum - Brain or cervical spine surgery in previous 5 years; trauma to head or neck requiring hospitalization in previous year - Severe, unmanaged depression

### Changes to the study protocol

Initially, participants with a history of migraine headaches in the last year were excluded. In the second year of the study, this criterion was modified to exclude participants who had >1 migraine headache day per month in the last year. The primary rationale for this change was to enhance recruitment and enrollment at both sites. The likelihood that this change is a major confounder is low due to the infrequent nature of included migraine headaches, the distinct nature of the symptoms [5], and study participants ability to recognize migraine and cervicogenic headaches as different. In addition, the analysis was modified to include stratification by the presence or absence of migraine headaches.

### Definition and diagnosis of cervicogenic headache

CGH is identified as a distinct classification of headache by the International Classification of Headache Disorders [5]. The diagnostic criteria used are shown in Table 2 [32]. Criterion D is not used to diagnose cervicogenic headache or determine enrollment in this study; this criterion is not relevant in prospective treatment studies.

**Table 2:** Cervicogenic headache diagnostic criteria (ICHD 2^nd^ Edition)

A.	Pain, referred from a source in the neck and perceived in one or more regions of the head and/or face, fulfilling criteria C and D.
B.	Clinical, laboratory and/or imaging evidence of a disorder or lesion within the cervical spine or soft tissues of the neck known to be, or generally accepted as, a valid cause of headache.
C.	Evidence that the pain can be attributed to the neck disorder or lesion based on at least one of the following:
	1. Demonstration of clinical signs that implicate a source of pain in the neck.
	2. Abolition of headache following diagnostic blockade of a cervical structure or its nerve supply using placebo- or other adequate control.
D.	Pain resolves within 3 months after successful treatment of the causative disorder or lesion.

Key: *ICHD* international classification of headache disorders

Participants undergo a thorough headache history at baseline to confirm a temporal sequence exists linking the headache to the neck. A manual exam of the cervical spine is conducted to identify areas of joint dysfunction [33]. Participants who meet these criteria, without symptoms suggestive of a different headache (e.g., prodromal aura, sensitivity to light and sound), are diagnosed with CGH.

### Eligibility determination

#### Phone screen

Potential participants respond to recruitment materials and trained interviewers conduct telephone screens to assess obvious eligibility criteria. Suitable candidates are then scheduled for the first of two baseline evaluation appointments within 60 days of the phone screen.

#### Baseline 1 evaluation

The first baseline evaluation (BL1) includes an in-person informed consent. Participants complete a self-report questionnaire, and undergo a health history, physical examination, and cervical radiographs, which are used to rule out contraindications to treatment (e.g., diffuse idiopathic skeletal hyperostosis, severe degenerative joint disease). Previous medical records are also obtained as needed to rule out contraindications. A trained and certified examiner performs a blinded, objective assessment of cervical spine motion, and static and dynamic pain pressure thresholds. Qualified participants at BL1 are scheduled 4 weeks later for the baseline 2 screening evaluation (BL2). In the interim, participants complete a daily headache diary to capture CGH frequency and intensity, medication use, and other types of headaches.

#### Case review meetings

Following BL1, study personnel review each case weekly to determine preliminary study eligibility. Patient safety and compliance concerns are also addressed. These meetings ensure consistent interpretation and application of the pre-defined eligibility criteria. Consensus is reached for all cases and recommendations for inclusion, exclusion, or follow-up care are made.

#### Baseline 2 evaluation and first treatment

Participants recommended for inclusion at case review return for the BL2 screening evaluation. This includes review of informed consent and completion of a self-report questionnaire. Headache diaries completed during the 4-week baseline period are collected, and eligibility is confirmed using checklists. Eligible patients are randomly assigned to one of 4 treatment groups by blinded study staff, receive their first of 18 treatments, and begin the second headache diary.

### Treatment allocation and concealment

An adaptive computer-generated rank-minimization scheme is used to allocate eligible participants to treatment [34]. The computer program balances 7 blocking variables separately for each of the 2 sites and is stratified by the presence of infrequent migraine headache: age, gender, CGH frequency and intensity, previous experience and confidence in SMT and professional massage therapy, and concomitant tension-type headache. Allocation concealment is protected by the use of balancing variables for rank minimization collected immediately prior to enrollment. Also, the number of participants previously randomized to each group is concealed from the study personnel involved in eligibility determination.

A computer generated random allocation sequence secured in sequentially numbered, opaque envelopes randomized within blocks is used to randomize the first several participants at each site to provide a “seed” group of participants for the dynamic allocation program. Envelopes are also used in the rare case of rank minimization program malfunction. Envelopes are created by the study statistician such that treatment allocation and block sizes are concealed from all study personnel.

### Interventions

Treatments are provided at university-affiliated clinics by licensed chiropractors with 6–35 years of clinical experience. At the Minnesota site, treatments are provided in a university research clinic. Treatments at the Oregon site are provided at one university clinic and 9 private chiropractic clinics. Participants commit to three, 10-min visits per week for 6 weeks (18 visits total), and the 10-min visits are standardized across sites. In the first 5 min, a moist hot pack is applied to relax neck and upper back musculature; cold packs are used for acute exacerbations. During this time, the chiropractor conducts a brief history, including subjective headache and/or neck complaints, patient progress, complications and medication changes. In the remaining 5 min, a standardized exam of the cervical and upper thoracic spine and musculature (occiput to 3^rd^ thoracic vertebra) is performed to identify sites of joint dysfunction to be treated. This includes manual palpation for segmental motion restriction, tenderness, and osseous asymmetry, as well as decreased global range of motion [35]. The study treatment is then delivered (Table 3). No other care is provided.

**Table 3:** Treatment interventions

Intervention	Spinal manipulation therapy	Light massage therapy
Type	High Velocity, low amplitude Low Velocity, low amplitude (older patients or acute exacerbations only)	Gentle effleurage (gliding) Gentle petrissage (kneading)
Location	Cervical spine (Occiput -C7) Thoracic spine (T1-T3)	Cervical spine musculature (Occiput -C7) Thoracic spine musculature (T1-T3)
Design & delivery format	Individualized: spinal levels treated, technique position (e.g., seated, supine, prone)	Individualized: site of pain/dysfunction
		Prone position
Delivery method	One-on-one treatment visit	One-on-one treatment visit
Dose	10 min 3 visits per week for 6 weeks	10 min 3 visits per week for 6 weeks

To minimize intervention bias and confounding by contextual effects introduced by the provider during care, chiropractors are trained to interact with patients with equal enthusiasm for treatments across groups. Data regarding patients’ perceptions of their provider’s enthusiasm and confidence in the assigned study care are collected on questionnaires during and immediately following treatment (week 6) [26].

Standardized forms are used to document treatment procedures and side effects, and all forms are reviewed for protocol deviations and adverse events. Participants are asked to refrain from seeking care for their CGH and neck pain from other providers during the baseline and treatment periods; however, abortive analgesics are permitted as needed. Outside care is unrestricted following the treatment phase; data regarding non-study care is collected on self-report questionnaires.

Treatment providers are trained and certified to ensure standardization in intervention and documentation across study sites. Quality assurance includes meetings with providers to review treatment protocols and discuss uniform enthusiasm for both interventions. Meetings occur quarterly in Year 1 and biannually thereafter to ensure uniformity of care. Quality control measures are implemented to ensure compliance with the study protocol. Specifically, treatment forms are reviewed for completion after each visit, and all clinicians are observed at predetermined intervals throughout the active treatment phase. Following investigator, or designee observations, providers are recertified monthly in Year 1 and quarterly thereafter. Further, participants’ perception of their clinicians’ enthusiasm for care is used to give feedback to clinicians if imbalances across treatment groups are found.

#### Spinal manipulation

Spinal manipulation therapy (SMT) consists of the diversified thrust maneuver as described by Peterson and Bergmann [35]. The need for SMT is assessed at each visit via patient progress and a manual cervical palpation exam [35]. The clinician documents the sites of dysfunction and manipulates some or all dysfunctional regions based on the patient’s tolerance. SMT is not performed if there are no signs of joint dysfunction (i.e., palpable joint pain and/or restriction) or if a new contraindication exists. Modifications to high velocity, low amplitude manipulations are made for older participants and those with acute exacerbations (i.e., low-velocity, low-amplitude mobilization) [35]. Other chiropractic techniques are not permitted.

#### Light massage

Light massage consists of gentle effleurage and pétrissage applied to the neck and shoulder muscles [35, 36]. Clinicians use massage lotion, focus on the site of pain and dysfunction, and gently squeeze tender points to reinforce the sensation of therapy. Manual trigger point therapy is not permitted. Dose–response trials require a manual comparison group to control for attention and therapeutic touch, in this case visits without spinal manipulation therapy. In a trial comparing SMT and light massage, a minimal light massage was credible to participants [29].

#### Treatment dose

Participants are randomized to one of four doses (*n* = 64 per group): 0, 6, 12, or 18 SMT visits. All participants are assigned 18 total visits. When participants do not receive the index therapy (SMT), they receive the control therapy (light massage).

### Data collection

Patient self-reported outcomes are collected at baseline and 6, 12, 24, 39, and 52 weeks post randomization using headache diaries and questionnaires. Objective spinal biomechanical outcomes and pain pressure thresholds are collected at BL1 and at 6 weeks (end of treatment). Qualitative interviews are also conducted at the end of treatment. Study data is managed using Research Electronic Data Capture (REDCap) tools hosted at the University of Western States [37].

#### Headache diary

The International Headache Society (IHS) recommends using a daily headache diary to capture headache outcomes in efficacy trials [38]. An electronic diary, the preferred method, is sent to participants using a Short Message Service (SMS) or email response (SMS-Track ApS, Denmark https://www.sms-track.com/). Paper diaries are provided to participants without daily Internet or mobile phone access and are used as a back-up when the electronic platform is not available. Enrolled participants complete 6 headache diaries: one at baseline (to determine study eligibility), one during the treatment phase, and four during the follow-up phase. Diary questions are found in Table 4.

**Table 4:** Diary questions

Diary questions	Response options
1. Did you take medication for a neck-related headache today?	1. Yes or No
2. Did you have a neck-related headache today?	2. Yes or No
3. If yes, rate your neck-related headache today?	3. 11-box numerical rating scale (0 = no pain, 10 = worst pain possible)
4. Did you have a headache not related to your neck?	4. Yes or No

Email or SMS options are available to participants depending on patient preferences. Automatic SMS messages (i.e., text-messages) or emails are sent to participants each night during the data collection period, and participants are given 24 h to respond. Participants who fail to respond during the designated time period cannot retroactively complete the diary. For those whose preference is email, 1 email is sent to the participant’s preferred address containing four questions (Table 4). Participants respond to the questions, submit their responses, and receive a thank you notification confirming successful delivery and data capture. Participants who chose the SMS option receive four individual SMS messages, one for each of the four questions. Paper diaries are mailed in advance.

### Outcomes

#### Primary aim: dose–response

##### CGH frequency

The primary outcome measure is self-reported CGH frequency, measured as the number of days with CGH in the 4 weeks prior to weeks 12 and 24 using a headache diary. The IHS guidelines for controlled trials recommend headache frequency as the most appropriate primary measure in efficacy trials [38]. Participants are also asked to recall the number of CGH days in the past 4 weeks on questionnaires, which are used as backup to the diaries. These time points were chosen to include short and long-term primary outcomes. CGH frequency measures at 6, 39, and 52 weeks are secondary outcomes (Table 5).

**Table 5:** Data collection schedule

Outcomes	Variables	BL1	BL2	2W	6W	12W	24W	39W	52W
Background	Demographics; comorbidities	Q							
	Duration of episode; history of SMT and massage care		Q						
Primary Objective	Dose-Response								
Headache	CGH frequency in 4 weeks prior	Q	D, Q		D, Q	D, Q	D, Q	D, Q	D, Q
	CGH pain	Q	D, Q		D, Q	D, Q	D, Q	D, Q	D, Q
	CGH disability		Q		Q	Q	Q	Q	Q
	CGH improvement: pain, global perceived effect				Q	Q	Q	Q	Q
	Other headache days		D, Q		D, Q	D, Q	D, Q	D, Q	D, Q
Neck Pain	Frequency (days) of neck pain	Q	Q		Q	Q	Q	Q	Q
Quality of Life	EuroQol-5D		Q			Q	Q	Q	Q
Satisfaction	Likert Scale, success of care				Q	Q	Q	Q	Q
Medication	Frequency of prescription and non-prescription medication (days)		D, Q		D, Q	D, Q	D, Q	D, Q	D, Q
Outside Care	CGH care from external providers		Q	Q	Q	Q	Q	Q	Q
Objective Measures	Kinematics and cervical segmental joint function	X			X				
Secondary Objective	Cost Effectiveness								
Healthcare Utilization and lost productivity			Q		Q	Q	Q	Q	Q
Utilities	Quality-adjusted life years EuroQol-5D		Q			Q	Q	Q	Q
Outcomes	CGH frequency (days) and intensity (in last 4 wks)	Q	D, Q		D	D	D	D	D
Tertiary Objective	Effects of Expectations								
Quantitative	Confidence in treatment success		Q	Q	Q	Q	Q	Q	Q
	Perceptions of the Patient-provider encounter			Q	Q				
Qualitative	Meaning, constructs of expectations				I				

Key: *BL* baseline, *SMT* spinal manipulation therapy, *W* weeks, *CGH* cervicogenic headache, *Q* questionnaire, *D* diary, *I* interview, *X* indicates occurrence

##### CGH pain

Patient-rated headache intensity is the principal secondary outcome and is measured using the valid and reliable 11-point numerical rating scale [39]. Intensity is the average of daily pain rated over four weeks on the headache diary. Diaries are backed-up by participants recall of their average pain over the last 4 weeks collected on the questionnaires using the same scale. Evaluation of headache pain is recommended by the IHS [38].

##### Disability

Patients rate their headache-related disability using the valid and reliable Headache Impact Test (HIT-6) [40]. This 6-item survey is administered at BL2 and following the intervention phase.

##### Quality of life

This is collected using the EuroQol-5D, a multi-attribute utility scale. The 5 dimensions (mobility, self-care, usual activities, pain/discomfort, and anxiety/depression) are evaluated with three levels (no, moderate or severe problem). This will be used to compute quality-adjusted life years for the cost-utility analysis. This is a commonly employed instrument with sound psychometric properties [41, 42].

##### Improvement

Patient-rated global improvement is determined by asking participants to compare their headache condition to what it was before study treatment on a 9-point ordinal scale ranging from no symptoms (100% improvement) to as bad as it could be (100% worse). Improvement has been shown to be reliable and responsive [43]. Improvement in CGH pain is also evaluated on a −10 to +10 numerical rating scale.

##### Other headache days

Participants indicate whether they have headaches other than cervicogenic on the headache diary. The sum of headache days in the last 4 weeks is used.

##### Neck pain frequency

Self-reported neck pain frequency, measured in days, is collected on the mailed questionnaires. Participants are asked if they had neck pain over the last four weeks (yes or no), and if so, how many days they had neck pain (1–28 days).

##### Neck pain

Participants are asked to rate their average neck pain over the past 4 weeks using the valid and reliable 11-box numerical rating scale (0 = no pain, 10 worse pain possible) [39]. Participants with spinal problems consider pain to be an important outcome measure [44].

##### Satisfaction

Participants overall satisfaction with treatment is measured using a 6-point Likert scale ranging from extremely dissatisfied to extremely satisfied. This scale is adapted from the Interstudy’s Low Back Pain TyPE Spec [45].

##### Medication use

Participants report frequency of over-the-counter and prescription medication use on diaries and questionnaires. The daily diary asks: “Did you take any medication for a neck-related headache today?” (yes or no).

##### Outside care

Discretionary professional care outside the study, including emergency care and hospitalizations rendered for CGH, is captured on questionnaires. Participants are asked, “In the past four weeks, have you seen any healthcare provider for your neck related headaches?” (yes or no). If yes, participants indicate which type of provider they visited and the frequency of visits.

##### Cervical kinematics

These are evaluated using the Zebris CMS-HS Spine Motion Analyzer (Zebris Inc., Isny im Allgau, Germany) and a modified protocol described by Wang [46]. This reliable and accurate tool has been used in other RCTs [46–49]. Flexion, extension, lateral flexion and rotation, coupled out of plane motion, and maximum accelerations and velocities in these planes are recorded.

##### Pressure pain threshold

These are evaluated using a hand-held algometer (Wagner Instruments. Force One™ Digital Force Gage. FDIX. Greenwich, CT, USA) applied to standardized locations along the cervical and upper thoracic spine. In addition, pain pressure thresholds are assessed at areas identified as clinically important by the examiner using manual palpation techniques [35]. Examiners are trained across sites to apply pressure at a rate of approximately 1kg/s until pain is elicited. This is a reliable method for ascertaining pain pressure thresholds [50] and is shown to be associated with outcomes [30].

#### Secondary aim: cost-effectiveness

Direct and indirect healthcare costs are included. Direct costs are determined from health services utilized and indirect costs from lost productivity [51].

##### Healthcare utilization

The utilization of health services for headache not provided in the study are captured on questionnaires at all-time points. Health services include physician and other provider visits, hospital services, prescription and non-prescription medication, advanced imaging, and classes/programs attended. Questions from the Community Tracking Study Household Survey [52] are used to capture provider and hospital services. Information for other service is ascertained using an instrument developed for a low back pain study [53]; this was also used in a CGH pilot study [29].

##### Lost productivity

Indirect costs, which include lost workdays and productivity, are assessed using three questions from the National Health Interview Survey [54]. Lost productivity data is captured on questionnaires.

#### Tertiary AIM: effects of expectations

##### Qualitative interviews

One-on-one (face to face), semi-structured qualitative interviews are conducted by trained research staff at week 6. An interview schedule is used to direct the interviews and keep interviewers on track with the study’s objective. Participants are asked about their treatment views, expectations and lived experience with headaches. Questions start broadly and are followed-up with probing questions to elicit additional information.

##### Confidence in treatment success

This is measured using a 7-point Likert scale adapted from the Interstudy’s Low Back Pain TyPE Spec [45]. Potential success is assessed at baseline and week 2. Confidence that assigned treatment is working is measured at all follow-up time points.

##### Perceptions of the patient-provider encounter

Perceptions are evaluated at weeks 2 and 6 and are used to determine the effect of the patient-provider experience on expectations and outcomes [26, 29]. The instrument used is based on the satisfaction questionnaire by Cherkin et al. [55, 56]. Specifically, participants are asked about their level of agreement with statements about their perception of the treating chiropractors’ adequate time spent listening, comfort with dealing with CGH, enthusiasm about the treatment program, and confidence that treatment will work.

### Blinding

Research staff who perform clinical and objective examinations and data entry are blinded to treatment assignment. Providers and participants are initially blinded to dose and the treatment hypotheses; however, treatment dose becomes apparent over time. Due to the nature of the SMT and light massage treatments, providers and patients are not blinded to treatments at each visit. Qualitative interviewers and project managers at both sites are not blinded.

### Adverse events

Incomplete reporting of adverse events remains a problem in clinical trials investigating therapies for CGH [57]. To address this, we use standardized and systematic recording and reporting of unanticipated and anticipated events. Enrolled participants are queried at each treatment visit and following the treatment phase (week 6) about problems, complications to care, or adverse events since their last appointment. During the follow-up data collection phase, participants are asked whether they spent >24 h in bed and/or if they were hospitalized for any reason on questionnaires; research staff contact participants by phone if an event occurred. Documentation of such events occurs on a standardized case report form which includes a severity rating, relatedness of the event to study participation, and whether the event was anticipated. Further, participants are asked to report serious adverse events to the study investigators or research staff. Oversight authorities (i.e., IRBs, funding agency) are informed about serious, reportable adverse events within three business days of the event first being identified. Non-reportable events are summarized for the data safety and monitoring board annually.

### Analysis plan

#### Primary aim: dose–response

##### Primary analysis

Both primary and secondary variables will be regressed on dose, baseline value of the outcome, site indicator (Portland or Minneapolis), and 6 baseline balancing variables used for randomization in the rank-order minimization computer program (CGH days, CGH pain, gender, age, tension-type headache indicator, and differential confidence in success of SMT and LM) [58]. The analysis will be stratified by migraine headache. Dose will be included in two separate ways: as a linear/nonlinear function and as a set of indicator variables to make comparisons between the SMT groups and the control group. Seemingly unrelated (simultaneous) regression by Zellner [59] will be used in the analysis for the individual time points [60]. For the primary outcomes only, longitudinal effects across all follow-ups will be modeled with generalized estimating equations to account for within-person correlation between time points [60]. An intention-to-treat analysis will be conducted with participants included in the original allocation group. Missing data will be imputed by interpolating between adjacent times, or by carrying forward when there is no subsequent data. An intention-to-treat analysis will be conducted with participants included in the original allocation group. Missing data will be imputed by interpolating between adjacent times, or by carrying forward when there is no subsequent data. This method was used in our pilot study [28] and full-scale, dose–response randomized trial on manipulation for low back pain [61] where the quantity of missing data was reasonably small. Sensitivity analyses 1) using multiple imputation and 2) excluding imputed data will be performed to assess the impact of missing data [62, 63].

##### Responder analysis

CGH days, pain, and disability, as well as neck pain will be dichotomized using a 50% improvement threshold to define a responder. This is a common measure of important improvement in headache research [38]. The dichotomized data will be analyzed using binomial regression models [60] with the same independent variables as the primary analysis to compare responder rates between groups.

#### Secondary aim: economic analysis

Incremental cost-effectiveness ratios (ICERs) will be computed using adjusted between-groups differences in costs (numerator) and effects (denominator). Incremental cost-utility ratios will be computed replacing the denominator with quality-adjusted life years (QALYs) derived from the EuroQol EQ-5D [41, 42]. Regression analyses will be used to model cost and effect separately to adjust each for potential confounding variables [64–66], with bias-corrected and accelerated non-parametric bootstrap used to address cost-data skewness [67]. Bootstrapped cost-effect pairs will be plotted on the cost-effectiveness plane to assess the amount of uncertainty surrounding ICER estimates. In addition, cost-effectiveness acceptability curves will be used to estimate the probability of cost-effectiveness over a range of willingness to pay thresholds [68].

The principal cost analysis will consider only the scheduled treatment visits defined in this protocol. A second approach will adopt a societal perspective including all healthcare resources [51, 69, 70] and estimated costs of lost productivity due to CGH [71, 72].

#### Tertiary aim: effects of expectation

##### Quantitative analysis

The potential effects of patient expectancy will be explored in a path analysis using structural equation modeling [73, 74] as in our earlier work [26, 75]. Dose effects will be included in a measurement model, and expectancy variables will be permitted to affect subsequent outcomes, as well as being influenced by previous outcomes. Due to the limited sample size, this analysis will be carried out as an exploratory analysis.

##### Qualitative analysis

For the qualitative interviews, content analysis using an inductive approach [76] will be used to identify categories and themes that occur in the transcribed text [77]. Transcribed data will be entered into a database designed to capture and analyze qualitative data. The frequency of themes will be quantified and representative quotations will be identified [77, 78]. The frequency of responses in the thematic categories will be cross-tabulated with treatment group assignment and compared for between group differences using Chi-square analysis. 95% confidence intervals will be calculated for these differences.

### Power analysis and sample size

A power analysis was conducted by simulating the primary analysis outlined above using the primary outcome, CGH days. We will randomize 256 participants (*n* = 64/group). Sample size is based on 80% power using a two-sided test at the .05 level of significance. For the primary outcome, CGH days, we will be able to detect a linear dose effect (slope) of 1.1 CGH days (between two adjacent doses) and a mean difference of 3.5 CGH days between 2 groups. The residual standard deviation of 7 headaches (surrogate for days) was used, based on regression analyses in the CGH pilot study [29]. The study will also be appropriately powered for CGH intensity, the principal secondary outcome.

### Data and safety monitoring plan

University of Western States is the data coordinating center responsible for the creation of case-report forms, data transfer and management. The data and safety monitoring board and 2 university institutional review boards meet annually to review the study. Reportable adverse events will be reviewed as they occur. No interim analyses are planned due to the low risk profiles of the interventions used in this study.

### Study status

Enrollment is complete at the Minnesota and Oregon sites. Follow-up data collection is ongoing thru 2016.

## Discussion

This novel study is the first full-scale randomized controlled trial aimed at identifying the dose of SMT to treat cervicogenic headache in an adult population. The long-term goal is to begin to set the clinical study standards to establish criteria that can be used to inform the decision-making process to 1) select optimal chiropractic care protocols for future comparative effectiveness trials and 2) determine the appropriate number, frequency, and duration of various chiropractic services for headache and other commonly treated neuromusculoskeletal conditions. Further, the use of a daily electronic headache diary to capture principal headache outcomes in real-time in both the short and long-term provides data arguably less subject to recall bias compared to other platforms such as paper diaries. To our knowledge, electronic diaries have not been used to collect headache outcomes in other CGH studies.

Headache is the most common pain condition resulting in lost work productivity, but cost data specific to CGH is lacking in the US [71]. Secondary outcomes used in this study will provide important information about the cost-effectiveness of SMT for CGH in adults. Further, several studies suggest a link between patient expectations and health outcomes [79–81]. However, in a pilot RCT, patient perception of the patient-provider interaction and patient expectations were balanced across groups and demonstrated little effect on outcomes [26]. To our knowledge, this is the first trial to use a qualitative and quantitative analysis to investigate the relationship of SMT dose to possible correlates including patient expectations, the patient-provider encounter, and headache outcomes. Information gathered will help to inform the design of future research and treatment regimens for patients with costly and prevalent headache conditions.

Notably, the fastidious nature of this trial and its methodologically rigorous design enhances internal validity. Specifically, the treatments are compared under tightly controlled conditions. Attention bias is controlled by standardized 10-min visits, equal number of visits, and restriction on concomitant care unassociated with the study. Patient expectation is controlled, in the absence of blinding, by equal enthusiasm for care by each chiropractor across treatment groups. The design also isolates the effects of manipulation by controlling the hands on time and attention from the chiropractor. In addition, strict eligibility criteria minimize the heterogeneity of the study population. To determine eligibility and ensure participant compliance, 2 baseline visits with a 4-week headache diary in between is strictly adhered to. Also, electronic diaries capturing headache outcomes are closely monitored and patients are reminded regularly to complete their diaries daily. To enhance generalizability, multiple recruitment strategies are used and participants are enrolled at 2 sites (West Coast and Midwest, USA).

Following completion of this trial, the study will provide dosing information and methodologically sound clinical evidence to inform the design of future comparative effectiveness trials and establish treatment protocols for patients suffering with CGH, a widespread and costly condition.

## Abbreviations

CGH, cervicogenic headache; CONSORT, Consolidated Standards of Reporting Trials; ICHD, International Classification of Headache Disorders; IHS, International Headache Society; IRB, institutional review board; LM, light massage; MN, Minnesota; OR, Oregon; RCT, randomized controlled trial; SMS, short message service; SMT, spinal manipulation therapy
